# Association between Kawasaki Disease and Autism: A Population-Based Study in Taiwan

**DOI:** 10.3390/ijerph110403705

**Published:** 2014-04-03

**Authors:** Ho-Chang Kuo, Chung-Min Wu, Wei-Pin Chang, Chun-Nan Kuo, Deniz Yeter, Chun-Yi Lin, Jei-Tsung Pai, Ying-Chen Chi, Chia-Hsien Lin, Liang-Jen Wang, Wei-Chiao Chang

**Affiliations:** 1 Department of Pediatrics, Kaohsiung Chang Gung Memorial Hospital and Chang Gung University College of Medicine, Kaohsiung 833, Taiwan; E-Mail: erickuo48@yahoo.com.tw; 2 Department of Business Management, National Taipei University of Technology, Taipei 106, Taiwan; E-Mail: cmwu@ntut.edu.tw; 3 Department of Healthcare Management, Yuanpei University, HsinChu 300 Taiwan; E-Mails: wpchang@mail.ypu.edu.tw (W.E.P.); elaine_lin76@ymail.com (C.Y.L.); reitrong@mail.ypu.edu.tw (J.T.P.); 4 Department of Clinical Pharmacy, School of Pharmacy, Taipei Medical University, Taipei 110, Taiwan; E-Mail: rencouter@gmail.com; 5 Shawnee, Kansas, 66226, USA; E-Mail: deniz.yeter@gmx.de; 6 Department of Education & Research, Taipei City Hospital, Taipei 106, Taiwan; E-Mail: a0130@tpech.gov.tw; 7 Department of Health Industry Management, School of Health Care Management, Kainan University, Taoyuan 338, Taiwan; E-Mail: g870615@gmail.com; 8 Department of Child and Adolescent Psychiatry, Kaohsiung Chang Gung Memorial Hospital and Chang Gung University College of Medicine, Kaohsiung 833, Taiwan; 9 Department of Pharmacy, Taipei Medical University-Wan Fang Hospital, Taipei 116, Taiwan; 10 Master Program for Clinical Pharmacogenomics and Pharmacoproteomics, School of Pharmacy, Taipei Medical University, Taipei 110, Taiwan; 11 Cancer Center, Kaohsiung Medical University Hospital, Kaohsiung Medical University, Kaohsiung 807, Taiwan

**Keywords:** Kawasaki disease, autism, population-based study, Taiwan population

## Abstract

*Objective*: The association between Kawasaki disease and autism has rarely been studied in Asian populations. By using a nationwide Taiwanese population-based claims database, we tested the hypothesis that Kawasaki disease may increase the risk of autism in Taiwan. *Materials and Methods*: Our study cohort consisted of patients who had received the diagnosis of Kawasaki disease (ICD-9-CM: 446.1) between 1997 and 2005 (*N* = 563). For a comparison cohort, five age- and gender-matched control patients for every patient in the study cohort were selected using random sampling (*N* = 2,815). All subjects were tracked for 5 years from the date of cohort entry to identify whether they had developed autism (ICD-9-CM code 299.0) or not. Cox proportional hazard regressions were then performed to evaluate 5-year autism-free survival rates. *Results*: The main finding of this study was that patients with Kawasaki disease seem to not be at increased risk of developing autism. Of the total patients, four patients developed autism during the 5-year follow-up period, among whom two were Kawasaki disease patients and two were in the comparison cohort. Further, the adjusted hazard ratios (AHR) (AHR: 4.81; 95% confidence interval: 0.68–34.35; *P* = 0.117) did not show any statistical significance between the Kawasaki disease group and the control group during the 5-year follow-up. *Conclusion*: Our study indicated that patients with Kawasaki disease are not at increased risk of autism.

## 1. Introduction

Kawasaki disease (KD) involves multisystemic vasculitis of unknown etiology. KD is a global disease and mainly affects children that are less than 5 years old, with the highest rate of incidence reported as being found in Asia, especially in Japan, Korea, and Taiwan [1,2,3]. The major clinical characteristics are prolonged fever more than 5 days, bilateral non-purulent conjunctivitis, diffuse mucosal inflammation with strawberry tongue, polymorphous skin rashes, indurative angioedema of the hands and feet, and unilateral non-suppurative cervical lymphadenopathy [2,4,5]. The most serious complications of KD are coronary artery aneurysm formation [4,6]. Both genetic factors and environmental factors have been considered as playing an important role in the prevalence of KD. 

In addition to the diagnostic criteria, there is a broad range of non-specific clinical features, including irritability, uveitis, aseptic meningitis, cough, vomiting, diarrhea, abdominal pain, gallbladder hydrops, urethritis, arthralgia, arthritis, hypoalbuminemia [7], liver function impairment, and heart failure [4,8,9]. From 1% to 30% of patients with KD exhibit central nervous system involvement, for example, aseptic meningitis, epileptic seizures, transient hemiplegia, facial palsy, ataxia, chorea, ischemia, hearing loss, abnormal vision, disturbed consciousness, and behavioral changes [10]. In addition, single photon emission computed tomography (SPECT) findings of KD patient with encephalitis/encephalopathy showed hypoperfusion of the bilateral cingulate gyri, thalamus, basal ganglia, brainstem, and cortex of the frontal lobes. All information indicated cerebral hypoperfusion. Vasculitis or cerebrovascular dehydration might be important factors [11].

Autism is a neurologic and developmental disorder that affects almost 1.1% of children in the United States [12] and 0.3% of children in Taiwan [13]. Children who have autism exhibit characteristic impairments in reciprocal social interaction, delayed and aberrant communication skills, and a restricted repertoire of activities and interests [14]. Recent study showed that autistic individuals have decreased cerebral perfusion, evidence of neuro-inflammation, and increased markers of oxidative stress [15]. Multiple independent SPECT and positron emission tomography (PET) studies revealed that hypoperfusion to several areas of the autistic brain, most notably over temporal regions and areas specifically related to language comprehension and auditory processing. Also several studies show that diminished blood flow to these areas correlates with many of the clinical features associated with autism. Hyperbaric oxygen therapy (HBOT) has been used in several cerebral hypoperfusion syndromes successfully including cerebral palsy, closed head injury, fetal alcohol syndrome and stroke [15]. HBOT was reported to be of benefit for a number of individuals with autism spectrum disorders (ASD) which possessed certain physiological abnormalities including cerebral hypoperfusion, mitochondrial dysfunction, inflammation and oxidative stress [16]. 

Ichiyama *et al.* [17] reported that six of 21 children’s SPECT imaging demonstrated localized cerebral hypoperfusion without abnormal neurological findings or clinical symptoms, and the follow-up SPECT and MRI approximately 1 month after the first SPECT revealed no abnormalities when compared with the acute stage of KD. Delay in the development of spoken language is a characteristic feature of autism. One study found that hypoperfusion of the left temporal speech areas was detectable after the age of 5 years in autistic individuals. This hypoperfusion was correlated quite highly with the diagnosis of autism [18]. Another study found the more severe the autistic syndrome, the lower of cerebral blood flow is in temporal lobe. They suggested that left superior temporal hypoperfusion is related to autistic behavior severity [19]. Therefore, cerebral hypoperfusion may be an important factor for autism development. Since cerebral hypoperfusion is also noted in some KD children, whether they will have higher probability developing autism or not is concerned. The association between KD and autism has rarely been studied in Asian populations. Therefore, we investigated the hypothesis that KD may increase the risk of autism in Taiwan utilizing a nationwide Taiwanese population-based claims database for our research.

## 2. Methods

### 2.1. Database

A single-payer compulsory National Health Insurance (NHI) program was implemented in Taiwan in 1995. By the end of 2009, approximately 99% of all 23,500,000 residents of the population in Taiwan had enrolled in the NHI program. All claims data are collected in the National Health Insurance Research Database (NHIRD) at the National Health Research Institutes (NHRI), Taiwan.

The Taiwanese NHIRD consist comprehensive health care data, including the all inpatients and outpatients visits, procedure codes, catastrophic illness files, and various data regarding drug prescriptions of the insured for 23.5 million residents. Our data that was used to perform the analyses conducted in this study was retrieved from the Longitudinal Health Insurance Database 2005 (LHID2005), a subset of NHIRD. The LHID2005 consists of all the original medical claims for 1,000,000 enrollee’s historical ambulatory data or inpatient care data under the Taiwan NHI program from 1996 to 2010, and the database was created and publicly released to researchers. 

The NHRI has claims that there are no statistically significant differences in age or sex between the randomly sampled group and all beneficiaries of the NHI program. The NHRI also claim that each subject’s original identification number is encrypted for the sake of privacy in the LHID2005 dataset. The encryption procedures were consistent between other datasets so that all claims data could be linked to obtain the relative medical data required to conduct this study. 

### 2.2. Study Population

We used a study cohort and a comparison cohort to examine the relationship between KD and autism.The study cohort consisted of KD patients aged less than 5 years old who were newly diagnosed with KD [ICD-9-CM: 446.1] between January 1997 and December 2005. The date of the initial diagnosis of KD was assigned as the index date for each KD patient. To improve data accuracy, the KD selection criteria required all cases ICD-9 code to be assigned by a pediatrician. We also built selection criteria for autism (ICD-9-CM code 299.0X) patients who were identified under an ICD-9 code that was assigned by a psychiatrist or pediatrician. Our study used a study cohort and a comparison cohort to examine the relationship between KD and autism. Each KD cohort patient was matched based on age, sex, and index year to five randomly identified beneficiaries without KD subjects to build the comparison cohort. Patients diagnosed with autism before or after the study period were excluded from both cohorts. We also identified relevant comorbidities, including allergic disease such as atopic dermatitis (AD, ICD-9-CM code 691.8, 692.9, 692), allergic rhinitis (AR, ICD-9-CM code 477.X) and bronchial asthma (BA) (BA, ICD-9-CM code 493.X).

### 2.3. Level of Urbanization

For our study of urbanization, all 365 townships in Taiwan were stratified into seven levels according to the standards established by the Taiwanese NHRI based on a cluster analysis of the 2000 Taiwan census data, with 1 referring to the most urbanized and 7 referring to the least urbanized. The criteria on which these strata were determined included the population density (persons/km^2^), the number of physicians per 100,000 people, the percentage of people with a college education, the percentage of people over 65 years of age, and the percentage of agricultural workers. Since Levels 4, 5, 6, and 7 contained only a few KD cases, they were combined into a single group, thereafter referred to as level 4. 

### 2.4. Statistical Analysis

All data processing and statistical analyses were performed by using the Statistical Package for Social Science (SPSS) software, Version 18.0 (SPSS, Chicago, IL, USA) and SAS version 8.2 (SAS System for Windows, SAS Institute Inc., Cary, NC, USA). Pearson *X*^ 2 ^test was used to compare differences in geographic location, and urbanization level of patients’ residences between the study and comparison groups. We also performed a survival analysis using the Kaplan-Meier method, and used the log-rank test to compare the survival distributions between the cohorts. The survival period was calculated for patients who suffered from KD until an occurrence of hospitalization, an ambulatory visit for autism, or the end of the study period (31 December 2010), whichever came first. After adjusting for urbanization level, region, and the comorbidities as potential confounders in the study, we performed Cox proportional-hazards analysis stratified by sex, age group, and index year to examine the risk of autism during the 5-year follow-up in both cohorts. Furthermore, we did stratify the analysis of the underlying status of allergic disease condition to investigate the association between KD and autism events. Hazard ratios (HRs) and 95% confidence intervals (CIs) were calculated to quantify the risk of autism. Only the results of the comparisons with a two-sided *P* value of less than 0.05 were considered to represent statistically significant differences.

## 3. Results

**Figure 1:**
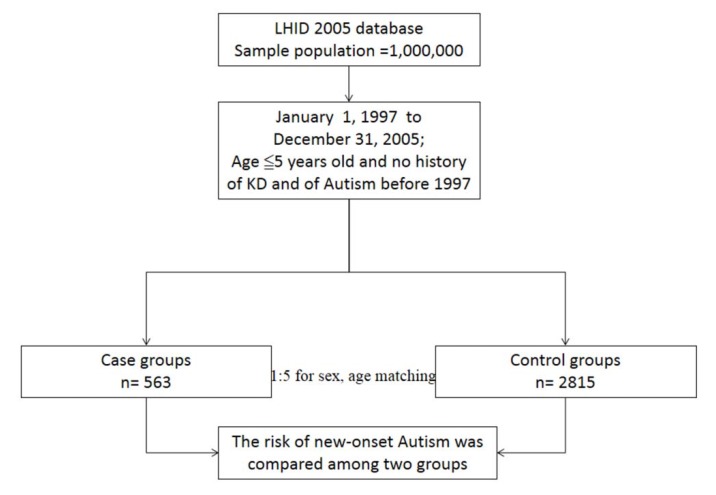
Flow chart of selection of study subjects and control subjects from the National Health Insurance Research database in Taiwan.

The research design of this study is detailed in Figure 1. The KD cohort contained 563 patients, and 2,815 patients were included in the comparison cohort. The distributions of demographic characteristics and the comorbidities for the KD and comparison cohorts are shown in Table 1. In comorbidities, bronchial asthma (*P* < 0.001), atopic dermatitis (*P* = 0.01) and allergic rhinitis (*P* < 0.001) were more prevalent in the KD cohort than the comparison cohort. In the KD group, we found that there was no statistical significant difference in urbanization (*P* = 0.11) and region variables (*P* = 0.08) as compared to the comparison-group subjects.

**Figure 2:**
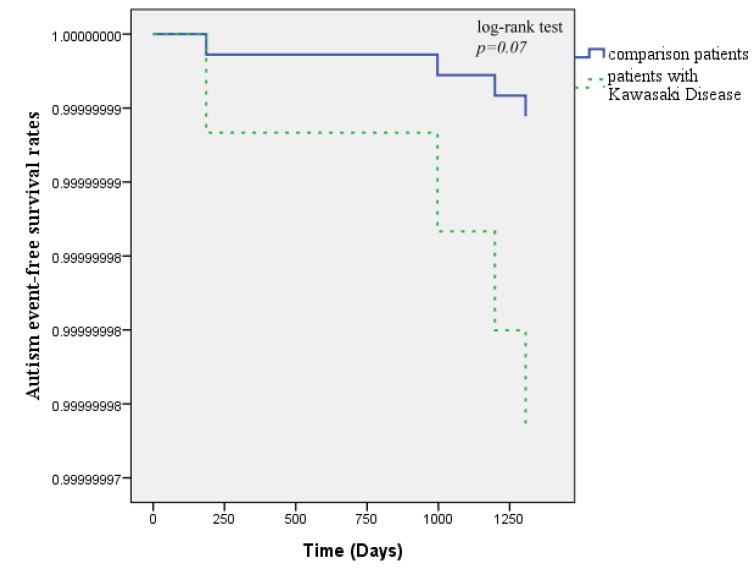
Autism-free survival rates for patients with Kawasaki disease and patients from the comparison groups from 1997 to 2005.

There were four patients noted to have autism during the 5-year follow-up, two patients with KD (0.4%) and two patients in the comparison cohort (0.1%) developed autism. The Kaplan-Meier survival curves are shown in Figure 2. The curves demonstrated that there were not significantly lower autism-free survival rates in the KD cohort than in the comparison cohort (log-rank test, *P* *=* 0*.*07). The overall incidence density was higher in the KD cohort (0.71 per 1,000 patient-years) than in the comparison cohort (0.14 per 1,000 patient-years).

Cox regression analysis showed that the crude HR of autism was 5.01 times greater for KD patients (95% CI = 0.71–35.58; *P* = 0.107) than for comparison patients. After adjusting for potential confounders (such as allergic factors), our results showed that newly diagnosed KD was not associated with autism (95% CI = 0.68–34.35; *P* = 0.117), as compared with non-KD patients (Table 2). 

We also stratified individual’s age into two categories: 0–2 years and 3–5 years. Table 3 demonstrated the risk developing autism is not different in the two groups (0–2 years of age group, HR: 4.37, 95% CI =0.27–70.34; *P* = 0.299; 3–5 years of age group, HR: 8.25, 95% CI = 0.47–144.65; *P* = 0.149).

**Table 1:** Demographic characteristics for the selected patients, stratified by presence/absence of Kawasaki disease from 1997 to 2005 (n = 3,378).

	Patients with Kawasaki Disease (n = 563)		Patients without Kawasaki Disease (n = 2,815)		*p* Value
	n	%	N	%	
Gender Male	341	60.6	1,705	60.6	1
Follow-up, year, mean (SD)	4.99	0.26	5.00	0.09	0.20
Urbanization level					
1 (most urbanized)	180	32.0	950	33.7	
2	170	30.2	713	25.3	0.11
3	91	16.2	469	16.7	
4 (least urbanized)	122	21.7	683	24.3	
Geographic region					
North	284	50.4	1,352	48.0	
Central	163	29.0	741	26.3	0.08
South	98	17.4	593	21.1	
Eastern	18	3.2	129	4.6	
	n	%	N	%	
Bronchial Asthma					
Yes	290	51.5	1,079	38.3	<0.001
No	273	48.5	1,736	61.7	
Atopic dermatitis					
Yes	436	77.4	2,037	72.4	0.01
No	127	22.6	778	27.6	
Allergic rhinitis					
Yes	417	74.1	1,761	62.6	<0.001
No	146	25.9	1,054	37.4	

**Table 2:** Hazard ratios (HRs) of autism among Kawasaki disease patients during the 5-year follow-up period from the index ambulatory visits or inpatient care from 1997 to 2005.

	Total		Patients with Kawasaki Disease		Patients without Kawasaki Disease	
Development of autism	NO.	(%)	NO.	(%)	NO.	(%)
5-year follow-up period						
Yes	4	0.12	2	0.36	2	0.07
No	3,374	99.88	561	99.64	2,813	99.93
Crude HR (95% CI)				5.01 (0.71–35.58)	1	
Adjusted HR (95% CI)				4.81 (0.68–34.35)	1	

Notes: Total sample number = 3,378. Both crude and adjusted HRs were calculated by Cox proportional hazard regressions, and stratified by age and sex. Adjustments are made for patients’ sex, age, asthma, atopic dermatitis, and allergic rhinitis. * Indicates *p* < 0.05;** Indicates *p* < 0.01; *** Indicates *p* < 0.001.

**Table 3:** Hazard ratios (HRs) of autism among Kawasaki disease patients and comparison cohort by age group.

Development of Autism	Age Group (Years)			
	0–2		3–5	
	Study group	Comparison	Study group	Comparison
	n (%)	n (%)	n (%)	n (%)
Yes	1 (0.25)	1 (0.05)	1 (0.64)	1 (0.13)
Crude HR (95% CI)	5.01 (0.31–80.04)	1	5.03 (0.32–80.41)	1
Adjusted HR (95%CI)	4.37 (0.27–70.34)	1	8.25 (0.47–144.65)	1

## 4. Discussion

The association of KD and autism has not been well established until now. Holmes *et al.* [18] revealed a statistically significant association between KD and the diagnosis of autistic disorder by Electronic Health Record (EHR) Systems and ADAMS, an Application for Discovering Disease Associations using Multiple Sources. Then we conducted the first population-based study in Asia, which has the highest incidence of KD by region in order to best investigate whether autism is truly a complication of KD. The results from this study showed no association in a population based study of Taiwan. 

From a review of the related literature, the long-term complications of autism as a result of KD or autistic individuals being more susceptible to KD have not been extensively described. Sato *et al.* [11] reported that SPECT findings of a patient with clinically mild encephalitis/encephalopathy with a reversible splenial lesion (MERS) is associated with KD, which showed hypoperfusion of the brain and indicated that the pathogenesis of MERS is based on cerebral hypoperfusion due to vasculitis or cerebrovascular dehydration. Ichiyama *et al.* [17] also reported that six of twenty one children’s SPECT imaging demonstrated localized cerebral hypoperfusion without abnormal neurological findings or clinical symptoms, and the follow-up SPECT revealed no abnormalities in acute stage of KD. The hypoperfusion of brain in KD patients were found only in acute stage patients and lasting less than one month and did not cause the long-term complication of autistic disorder. 

Allergy induced autism is an area of research wherein immune responses to some allergens may play a pathogenic role in autism [19]. Several recent reports revealed that KD patients were at an increased risk for allergic diseases including asthma [20], allergic rhinitis and atopic dermatitis [21,22,23,24]. Allergy may be a contributing factor to the increased serum levels of anti-myelin basic protein (anti-MBP) and anti-myelin associated glycoprotein (anti-MAG) auto-antibodies in some autistic children. Indeed, further study for the links between allergy, the immune system and the brain in cases of autism is needed.

Urbanization also supports that autism did not increase after KD being diagnosed. Increased risk for autism with increasing degree of urbanization has been identified as a significant factor in multiple geographically and ethnically diverse areas including Japan [25], Denmark [26], and the United States [27]. This may suggest that potentially increased autism risk as a result of urbanicity is not ethnically specific but may be more directly related to urbanicity itself. Regardless, urbanization does not appear to be an important effect modifier of KD [28].

It is noteworthy that autistic spectrum disorders encompass a broad spectrum ICD9-CM 299.X of diagnosis. To ensure the patients with autistic spectrum disorders as a homogeneous group, we only recruit patients with the diagnosis of autism (ICD-9-CM code 299.0X). However, the definition of autism in this study solely based on ICD code that appeared once in database, and the diagnosis of autism were not validated. The findings should be further verified by a clinical cohort study in the future. In addition, if children who were suspected to have developmental delay or mental disorders in school in Taiwan, the teachers often suggest children’s caregivers to take their children visiting the out-patient department of child psychiatry. Nevertheless, not every caregiver accepts the suggestion. By contrast, KD is a chronic illness, and patients with KD require regular follow-up in hospitals. Their families might have greater accessibility to medical information and as a result a greater awareness about patients’ health conditions than the comparison group. Therefore, this issue might also influence the opportunity to meet child psychiatrists and could be diagnosed as having autism.

There are limitations to this study that are worth noting. First, environmental and genetic risk factors, which could affect the susceptibility of autism, were not included in our analysis. Second, household income and economic status could be important factors for the evaluation of medical access or medical utilization. Third, family history should be considered to further confirm the association between autism and KD in the Taiwanese pediatric population. Last, diagnosis of autism could be a difficult and time-consuming process [29]. This study used reimbursement data, and the diagnoses of autism were not validated using structural diagnostic instruments. Furthermore, according to the Diagnostic and Statistical Manual of Mental Disorders, Fourth Edition (DSM-IV-TR) criteria [14], delays or abnormal functioning typically onset prior to age 3 years in children with autism. Clinicians might not necessarily make a diagnosis of autism, while they saw a patient whose first autism-like symptoms exhibited older than 3. Despite these limitations, the use of a nationwide dataset, which provides a large sample size, enabled us to evaluate the relationship between KD and the risk of developing autism during the 5-year follow-up period.

In conclusion, our results provide evidence that KD is not associated with the prevalence of autism in Taiwan. To better explore these two diseases, future studies that also involve social mechanisms and lifestyles are required.

## 5. Conclusions

The findings of our population-based study indicate that patients with KD are not at increased risk of autism. 

## Abbreviations

KD: Kawasaki Disease
IVIG: Intravenous Immunoglobulin
BA: Bronchial Asthma
AS: Atopic Dermatitis
AR: Allergic Rhinitis
